# *Mycobacterium bovis* Infection, Lyon, France

**DOI:** 10.3201/eid1209.060209

**Published:** 2006-09

**Authors:** Sophie Mignard, Catherine Pichat, Gerard Carret

**Affiliations:** * Centre Hospitalier Lyon Sud, Lyon, France;; † Centre National de la Recherche Scientifique, Lyon, France

**Keywords:** *Mycobacterium bovis*, human infection, molecular typing, BCG, dispatch

## Abstract

In a 5-year retrospective study, we used spoligotyping and mycobacterial interspersed repetitive units to type 13 strains of *Mycobacterium bovis* isolated from human sources. Despite the relatively high incidence of human tuberculosis caused by *M. bovis* (2%), these tools showed no clonal evolution and no relationships between the isolates.

*Mycobacterium bovis* belongs to the *M. tuberculosis* complex (MTBC) and has a wide host range, infecting animals and occasionally humans. *M. bovis* has been a historical source of tuberculosis (TB) in humans infected through drinking contaminated unpasteurized milk or inhaling aerosols produced by diseased farm animals. Due to a national program of TB control, the incidence of *M. bovis* in France has dramatically decreased in cattle herds, falling from 10% in the1960s to 0.09% in 1998, and in humans, falling from 1.5% of TB cases in the 1960s to 0.5% (0.07/100,000) in 1995 (*1**,**2*). We describe 13 (2 were BCG strains) of 555 MTBC strains isolated from human samples (2% of incidence; we did not quantify the BCG strains), in Lyon, France, over a period of 5 years. Despite the small number of patients, our study shows a relatively high local incidence of infections caused by *M. bovis*. Advances in molecular typing have improved our understanding of the dissemination of *M. bovis* and helped improve our ability to distinguish among strains. Spoligotyping and mycobacterial interspersed repetitive units–variable number of tandem repeats (MIRU-VNTR) are now considered standard alternative molecular techniques (*3**,**4*). Both are PCR-based techniques that evaluate the polymorphism of the tandem repeat copy number at several loci and have been used to identify different strains of *M. bovis* (*5**,**6*). We used these molecular methods to identify different strains of *M. bovis*.

## The Study

From 2000 to 2005, positive cultures were obtained from 13 patients with a diagnosis of *M. bovis* infection. The strains were screened by using *pncA* gene for resistance to pyrazinamide sequencing, and all displayed the 169 C→G mutation (*7*). To differentiate between *M. bovis* and *M. bovis* BCG, we tested for the presence or absence of the region of difference 1 because the absence of this region is a specific marker of BCG strains (*7**,**8*). Spoligotyping was performed in accordance with Kamerbeek guidelines, and the data were compared with the Institute Pasteur (IP) Spoligotype Database and with the International *M. bovis* Spoligotype Database (*9**,**10*). We performed MIRU-VNTR typing as described by Supply et al. (*11**,**12*).

Patient age, sex, sample site, and country of birth are provided in Table 1. Most of the clinical samples were from lymph nodes (n = 6). Others samples were from urine (n = 2), lung (n = 1), sputum (n = 1), cerebrospinal fluid (n = 1), ascitic fluid (n = 1), and synovial fluid (n = 1). Patient SO, who was 4 years old when his condition was diagnosed, had been born in France, but he spent months in Algeria with his grandmother who was ill with TB. Patient GD had a history of BCG-disseminated infection after being vaccinated with a BCG strain when he was 1 year of age. His condition had also been diagnosed as a familial form of septic granulomatosis, and he was immunocompromised. The strain was isolated only after he underwent lymph node resection at the age of 17. The bacillus isolated was an *M. bovis* BCG strain. Patient BL had undergone immunotherapy with a BCG strain for bladder cancer, and a BCG infection of the bladder developed.

**Table 1:** Patient data, *Mycobacterium bovis* infection, Lyon, France, 2002–2005*

Name	Age, y	Sex	Sample source	Country of birth
SO	4	M	Cervical lymph node	France
GD	17	M	Cervical lymph node	France
BS	35	F	Lymph node	Algeria
MB	36	M	Cervical lymph node	Djibouti
KA	38	M	Mediastinal lymph node	Morocco
GA	53	F	Urine	Algeria
PC	53	M	CSF	France
TG	54	M	Synovial fluid	France
FJ	59	F	Cervical lymph node	France
OM	71	M	Lung biopsy	France
GA	73	M	Ascites fluid	France
BL	78	M	Urine	France
RM	89	M	Sputum	France

*CSF, cerebrospinal fluid.

The results of spoligotyping and MIRU are shown in Table 2. Spoligotype profiles were typical of *M. bovis* with the absence of spacers 3, 9, 16, and 39–43 (*1**,**13*). Four distinct patterns were identified; the main one corresponded to spoligotype 482 in the IP database (70% of strains); both BCG strains exhibited this pattern. Others patterns represented were spoligotype 481 (2 strains) and 2 that were not included in the IP database (although one was identified as SB0914 in the international spoligotype database). These 2 spoligotypes (481 and 482) have been reported by Haddad et al. as the ones most commonly seen in bovine TB in France (*1*). Patient MB's spoligotype was not found in the databases, likely because of its origin (this patient was born in Djibouti), and it could be native to Africa.

**Table 2:** Spoligotyping and MIRU typing results

Patient	Strain	Spoligotype IP*	International spoligotype†	MIRU type‡	Spoligotypes
SO	*M. bovis*	481	SB0121	23232 42533 22	■■□■■■■■□■■■■■■□■■■■□■■■■■■■■■■■■■■■■■□□□□□
GD	*M. bovis* BCG	482	SB0120	22232 42533 22	■■□■■■■■□■■■■■■□■■■■■■■■■■■■■■■■■■■■■■□□□□□
BS	*M. bovis*	482	SB0120	23232 42512 22	■■□■■■■■□■■■■■■□■■■■■■■■■■■■■■■■■■■■■■□□□□□
MB	*M. bovis*	Not present	Not present	23232 42533 22	■■□□□□□■□■■■■■■□■■■■■■■□□□■□□□□□□□□□□■□□□□□
KA	*M. bovis*	482	SB0120	22232 52523 22	■■□■■■■■□■■■■■■□■■■■■■■■■■■■■■■■■■■■■■□□□□□
GA	*M. ovis*	481	SB0121	23232 42333 22	■■□■■■■■□■■■■■■□■■■■□■■■■■■■■■■■■■■■■■□□□□□
PC	*M. bovis*	482	SB0120	23232 42423 22	■■□■■■■■□■■■■■■□■■■■■■■■■■■■■■■■■■■■■■□□□□□
TG	*M. bovis*	482	SB0120	23232 42523 22	■■□■■■■■□■■■■■■□■■■■■■■■■■■■■■■■■■■■■■□□□□□
FJ	*M. bovis*	482	SB0120	22232 42523 22	■■□■■■■■□■■■■■■□■■■■■■■■■■■■■■■■■■■■■■□□□□□
OM	*M. bovis*	Not present	SB0914	23242 42533 22	■■□■■■■■□□□■■■■□■■■■■■■■■■■■■■■■■■■■■■□□□□□
GA	*M. bovis*	482	SB0120	23202 42523 22	■■□■■■■■□■■■■■■□■■■■■■■■■■■■■■■■■■■■■■□□□□□
BL	*M. bovis* BCG	482	SB0120	21232 42533 22	■■□■■■■■□■■■■■■□■■■■■■■■■■■■■■■■■■■■■■□□□□□
RM	*M. bovis*	482	SB0120	22231 43533 22	■■□■■■■■□■■■■■■□■■■■■■■■■■■■■■■■■■■■■■□□□□□

*IP, Pasteur Institute Spoligotype Database; available from http://www.pasteur-guadeloupe.fr/tb/spoldb3/spoldb3.pdf †International Spoligotype Database; available from http://www.Mbovis.org (*10*). ‡Mycobacterial interspersed repeat units (MIRU) type described by Supply et al. (11; available at http://www.ibl.fr/mirus/mirus.htm).

MIRU typing identified 12 individual patterns; 2 strains possessed the same MIRU patterns but not the same spoligotype. Both BCG strains showed the same pattern, except at locus 4 (*14*). Patient BL was found to have a BCG strain with 1 copy on locus 4. This profile is very similar to that of the Connaught strain used for the treatment of bladder cancer, which also has 1 copy at locus 4. Patient GD's strain of BCG had 2 copies at locus 4. This characteristic is similar to that of the BCG strain used for human vaccination in France (Mérieux strain derived from the Glaxo 1077 strain) (*14*).

## Conclusions

This 5-year study of human *M. bovis* infections in humans leads to 3 main conclusions. First, we observed a relatively high incidence of this disease: 2% of TB cases were caused by *M. bovis*, compared with 0.5% reported 10 years earlier and ≈1% reported in England by Smith in 2004 (*15*). Second, in France TB caused by MTBC occurs mainly in patients born abroad (55%), whereas in this study 70% of TB due to *M. bovis* occurred in French-born patients (4 of the patients had been born abroad). Therefore, human disease due to *M . bovis*, in contrast with that due *to M. tuberculosis*, appears to be predominantly indigenous in France, according to our study. However, we must note that human *M. bovis* infection varies throughout the world, even in industrialized countries, as reported in MMWR in 2005 when patients infected in New York were young persons born in Mexico or children of Mexican-born parents (*16*). Finally, we should note that French patients with *M. bovis* infections, in contrast to patients born abroad, were usually >50 years of age and sought treatment for a torpid infection. Measures to reduce bovine TB and the human transmission of *M. bovis* began in the 1950s. The disease was due to the reactivation of a past infection that had been acquired before milk pasteurization rather than a primary infection. Few cases have been reported in French-born children, which is in accordance with the effectiveness of preventive measures and their long-term effect. We cannot tell whether this is an emerging or a reemerging disease, but *M. bovis* is clearly still responsible for human TB. Global monitoring is required to confirm the progress of the disease and perhaps to explain why it is (re)emerging. In summary, we found the combination of spoligotyping and MIRU-VNTR to be a useful tool for identifying *M. bovis* infections and for determining whether patients were infected with the same strain. In our population of patients in Lyon, France, we did not detect any clonal epidemiologic features for *M. bovis* disease.
